# Functional modulation of human delta opioid receptor by neuropeptide FF

**DOI:** 10.1186/1471-2202-6-21

**Published:** 2005-04-04

**Authors:** Minna-Liisa Änkö, Pertti Panula

**Affiliations:** 1 Department of Biology, Åbo Akademi University, Tykistökatu 6A, 2nd floor, FI-20520 Turku, Finland; 2 Neuroscience Center and Institute of Biomedicine/Anatomy, University of Helsinki, Haartmaninkatu 8, FI-00014 University of Helsinki, Finland

## Abstract

**Background:**

Neuropeptide FF (NPFF) plays a role in physiological pain sensation and opioid analgesia. For example, NPFF potentiates opiate-induced analgesia and the delta opioid receptor antagonist naltrindole inhibits NPFF-induced antinociception. The nature of the interactions between NPFF and opioid receptors seems to be complex and the molecular mechanisms behind the observed physiological effects are not known.

**Results:**

We used a stable Chinese hamster ovary cell line expressing c-MYC-tagged human delta opioid receptor to study the interactions at the molecular level. Our results imply that NPFF can directly modulate the activation of delta opioid receptor in the absence of NPFF receptors. The modulatory effect, though only moderate, was consistently detected with several methods. The agonist-induced receptor trafficking was changed in the presence of (1DMe)NPYF, a stable NPFF-analogue. (1DMe)NPYF enhanced the receptor activation and recovery; opioid antagonists inhibited the effects, indicating that they were delta opioid receptor-mediated. The binding experiments with a novel ligand, Terbium-labeled deltorphin I, showed that (1DMe)NPYF modulated the binding of delta opioid receptor ligands. The levels of phosphorylated mitogen-activated protein kinase and intracellular cAMP were studied to clarify the effects of NPFF on the opioid signaling mechanisms. Application of (1DMe)NPYF together with a delta opioid receptor agonist enhanced the signaling via both pathways studied. Concomitantly to the receptor trafficking, the time-course of the activation of the signaling was altered.

**Conclusion:**

In addition to working via indirect mechanisms on the opioid systems, NPFF may exert a direct modulatory effect on the delta opioid receptor. NPFF may be a multi-functional neuropeptide that regulates several neuronal systems depending on the site of action.

## Background

Neuropeptide FF (NPFF) belongs to a family of RFamide peptides and was originally isolated from bovine brain [1-3]. It has a wide range of functions, including effects on pain mechanisms [1,4], opioid tolerance [5], cardiovascular regulation [6] and neuroendocrinological function [7].

At the physiological level NPFF seems to have both a direct analgesic effect and a modulatory effect on the opioid system. Some of the effects may be mediated via the NPFF receptors as two such receptors, NPFF1R and NPFF2R, have been identified [17-19]. Both NPFF1R and NPFF2R are expressed in the central nervous system and NPFF binds to both of them [17-20]. Also the other RFamide peptides bind to the NPFF receptors with varying affinities [21], and therefore the exact nature of the receptor-ligand interactions between RFamides and their receptors is still unclear.

The interaction between NPFF and opioid system in pain and analgesia seems to be complex in nature and the molecular mechanisms behind the observed physiological effects are not known. Binding studies have shown that NPFF does not displace opioid receptor ligands from any of the opioid receptor subtypes and opiates do not bind to NPFF binding sites [16]. However, many studies suggest that NPFF mechanisms are functionally coupled to the opioid system [for a review see ref. [8]]. In the rat spinal cord, the highest NPFF-like immunoreactivity is found in the superficial layers of the dorsal horn, an area involved in the nociceptive processes and pain mechanisms [9-11]. NPFF has been designated as a morphine modulatory peptide since it is able to influence the actions of opioid peptides within spinal cord and brain [8,12,13]. NPFF displays both anti-opioid and opioid-like effects depending on the route of administration. Supraspinal administration of NPFF attenuates opioid antinociception [1] and precipitates opioid withdrawal syndrome [5]. Intrathecally administered NPFF causes long-lasting analgesia, which is reduced by both naloxone and naltrindole [4]. NPFF in the periaqueductal grey produces a selective attenuation of tactile allodynia in neuropathic rats [14] that could be mediated indirectly by naloxone-sensitive opioid mechanisms [15]. In pontine parabrachial nucleus NPFF modulates synaptic transmission through interaction with presynaptic DOR, providing evidence for the cellular mechanisms of the analgesic action of NPFF at the supraspinal level [12].

Delta opioid receptor (DOR) belongs to the family of G-protein coupled, seven trans-membrane receptors [22,23]. DOR couples to the pertussis toxin -sensitive G_i/o_-type of heterotrimeric G-proteins. The receptor can regulate several effector systems, including adenylyl cyclase activity [22,24], the phosphorylation of mitogen activated protein kinases (MAPK) [25], voltage-gated calcium and potassium channels [26] and phospholipase C [27]. In CHO-cells the DOR-induced activation of MAPK-pathway is predominantly mediated by the Gβγ-subunit of G_i/o _[28] whereas adenylate cyclase response is mediated by the Gα_i/o_-subunit [24]. The involvement of DOR in analgesia has been shown using many pain models [29] and agonists acting at DOR have a strong antinociceptive effect [30]. The agonist stimulation causes rapid desensitization of the receptor by phosphorylation [31], which in turn produces the uncoupling of the receptor from its G-protein. The phosphorylation can be followed by endocytosis of the ligand-receptor complex [32] but the desensitization may occur also without receptor internalization [31]. The internalized receptor is either degraded or recycled back to the cell membrane [32,33].

We constructed a stable Chinese hamster ovary cell line expressing c-MYC-tagged human DOR (MYChDOR). The cell line was used as a model system to learn about the mechanisms involved in the interactions between NPFF and hDOR at the cellular and molecular level.

## Results

### Characterization of the cell line

**Figure 1:**
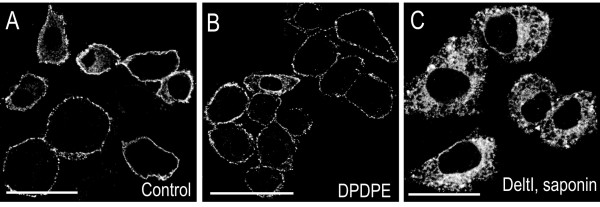
**Characterization of the CHO-K1 cells stably expressing MYC-tagged human DOR. A. **The cell surface receptors were visualized with an anti-MYC antibody and a fluorescent secondary antibody. **B. **The cells were treated with 100 nM DPDPE for 10 min at +37°C after which the remaining cell surface receptors were visualized with an anti-MYC antibody and a fluorescent secondary antibody. **C. **The cells were stimulated with 100 nM DeltI for 5 min at 37°C, permeabilized with saponin and the receptors were visualized with an anti-MYC antibody and a fluorescent secondary antibody. All the images are 0.5 μm confocal sections at the mid-nuclear level. Representative images are shown. Scale bar 20 μm.

The cellular localization of the expressed c-MYC-tagged delta opioid receptors in the stable CHO-K1 clone was analyzed with immunocytochemistry. The cells were stained with an anti-c-MYC antibody, detected with a fluorescent secondary antibody and analyzed with a laser scanning confocal microscope. The untransfected wild type cells did not show any fluorescence when stained similarly as the CHO/MYChDOR cells and the pre-absorption of the antibody solution with the c-MYC-peptide before immunocytochemistry removed all signal in the CHO/MYChDOR cells, implying that the antibody bound specifically to the MYC-tagged receptor (data not shown). In the CHO/MYChDOR cells a clear fluorescence signal was seen on the cell surface (Figure 1A) and DOR agonists induced the internalization of the receptor (Figure 1B &1C).

The receptor density and ligand binding characteristics of the expressed receptor were determined with ^3^H-diprenorphine binding assay. The observed values for K_D _and B_MAX _were 0.203 ± 0.090 nM and 0.530 ± 0.042 pmol/mg protein (318000 receptors/cell), respectively. The functionality of cells expressing MYC-tagged DOR was shown with the inhibition of forskolin-stimulated accumulation of cAMP and phosphorylation of ERK2. DPDPE dose-dependently inhibited the forskolin-induced accumulation of cAMP and similarly increased the phosphorylation of the ERK2 (data not shown and see below).

**Figure 2:**
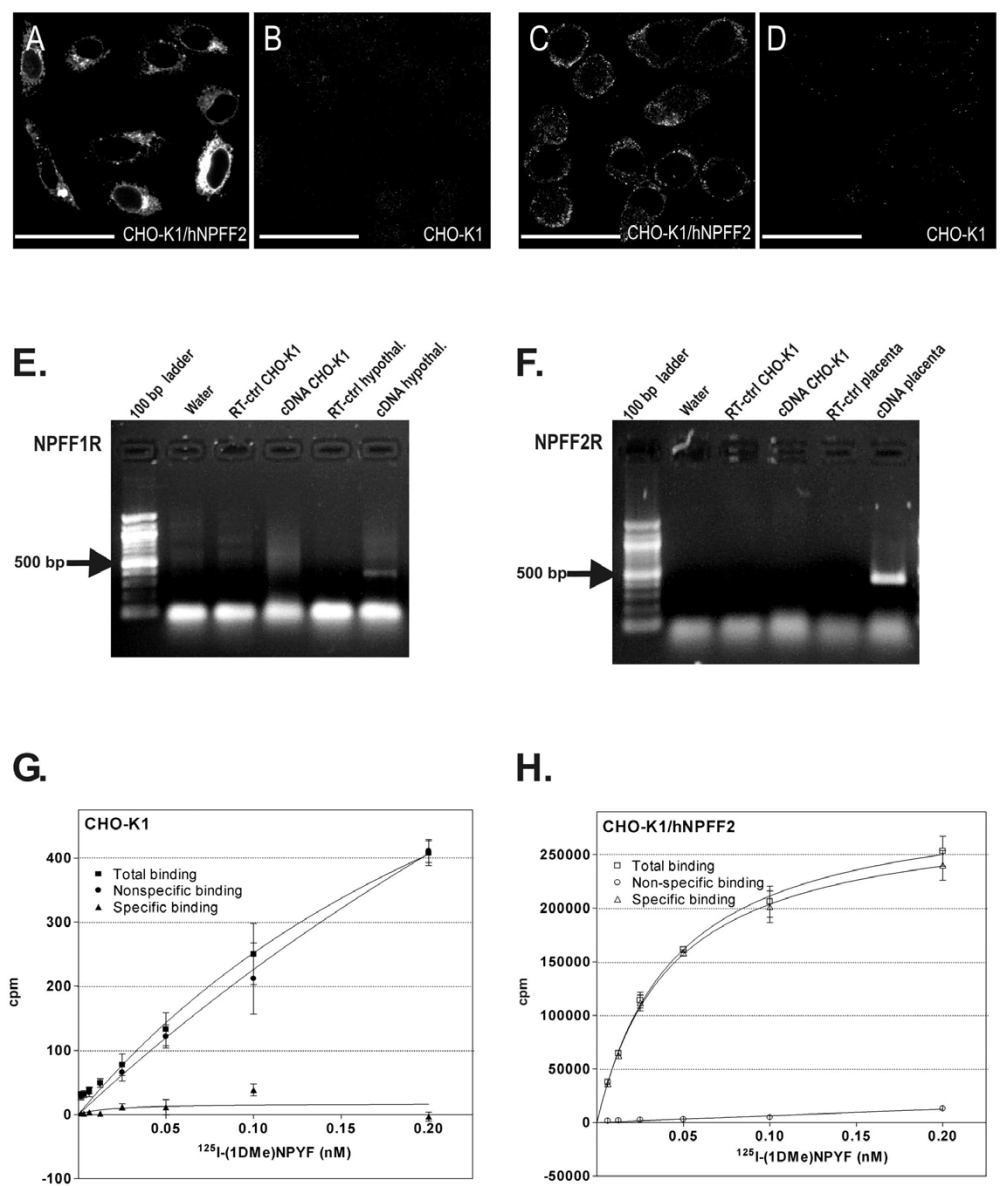
**CHO-K1 cells do not express NPFF receptors. **The presence or absence of NPFF receptors was analyzed with immunocytochemistry, RT-PCR and radioligand binding assay. **A. **CHO-K1 cells expressing hNPFF2R showed intense fluorescence when immunolabeled with an anti NPFF2R antibody detecting the C-terminus of the receptor. **B. **In the same conditions, the C-terminally oriented antibody did not label nontransfected CHO-K1 cells. **C. **The NPFF2R expression was also studied with an anti-NPFF2R antibody that binds to the N-terminus of the receptor. Again, in CHO-K1 cells expressing hNPFF2R immunoreactivity for the receptor was found. **D. **The N-terminally oriented NPFF2R antibody did not detect NPFF2R immunoreactivity in the nontransfected CHO-K1 cells. All the images are 0.5 μm confocal sections at the mid-nuclear level. Representative images are shown. Scale bar 20 μm. RT-PCR analysis gave further support for the absence of the NPFF receptors in CHO-K1 cells. **E. **cDNA from CHO-K1 cells were analyzed with hNPFF1 receptor specific primers and no PCR-product was obtained; human hypothalamus was used as a positive control (expected size of the PCR-product ~350 bp). **F. **The RT-PCR analysis of CHO-K1 cDNA did not give any PCR-product with hNPFF2R specific primers either; human placenta was used as a positive control (expected size of the PCR-product ~450 bp). **G. **CHO-K1 cells did not bind the NPFF receptor-specific radioligand at the concentration range where the positive control cell line expressing hNPFF2R showed saturable binding. The solid squares represent the total binding, the solid circles the nonspecific binding and the solid triangles the specific binding to the CHOK1 cell membranes. **H. **The cell membranes from CHO/hNPFF2R cells were used as a positive control in the experiment. The open squares represent the total binding, the open circles the nonspecific binding and the open triangles the specific binding to the cell membranes.

The possible presence of NPFF1R and NPFF2R in the CHO-K1 cells was studied with several methods. First the cells were studied with immunocytochemistry and confocal microscopy. Several antibodies against NPFF receptors were used. In the Figure 2A–D the results obtained with two antibodies are shown. The pre-absorption of the antibody solutions with the antigenic peptide before immunocytochemistry removed all immunoreactivity from CHO-K1/hNPFF2R cells in the case of each antibody tested, implying that the antibodies recognized specifically the antigen. No immunoreactivity was found in the untransfected CHO-K1 cells (Figure 1B &1D), which further suggested that the antibodies bound specifically to the hNPFF2R. Next the cells were analyzed with RT-PCR. cDNA made of total RNA from human medial hypothalamus and human placenta were used as positive controls for NPFF1R and NPFF2R, respectively, since a relatively high level of receptor expression has been shown in these tissues [17-20]. NPFF1R and NPFF2R specific primers based on human and mouse receptor sequences were used in the analysis. No PCR products were obtained from the CHO-K1 cDNA using conditions where the controls tested positive (Figure 1E &1F). The binding of NPFF receptor specific radioligand ^125^I-(1DMe)NPYF to the CHO-K1 cells was tested. The cells did not bind the ^125^I-(1DMe)NPYF (Figure 2G). Cell membranes of a commercial hNPFF2R expressing CHO-K1 cell line (Euroscreen S.A., Brussels, Belgium) were used as a positive control in the experiment (Figure 2H). ^125^I-(1DMe)NPYF bound to these cell membranes with the same affinity as reported by the supplier and as observed previously in our laboratory [21]. In addition, the NPFF-analogue alone did not affect any of the tested parameters, such as cAMP or MAPK (see below), which further supported the absence of NPFF receptors in CHO-K1 cells.

### Effect of (1DMe)NPYF on the agonist activated DOR trafficking

The CHO-K1 cells expressing MYC-tagged DOR were treated with 100 nM DPDPE (or DeltI) with or without 100 nM (1DMe)NPYF for 0, 5, 10 or 30 min at 37°C. The receptor trafficking was analyzed with fluorescence associated cell sorting, FACS. After the drug treatments, the cell surface receptors were detected with an anti-MYC-antibody and a fluorescent secondary antibody. At the basal state the amount of the cell surface fluorescence, which should reflect the amount of DORs located on the cell membrane, was observed to be variable between runs. To control the variation the cells were cultured for a fixed period of time after the last passage prior to internalization studies and grown into 80–90 % confluence. The cells were let to recover at 37°C after they were collected from the culture plates and before they were used for the experiments, to ensure that the receptor number on the cell membrane was balanced. Some constitutive receptor trafficking was still observed and therefore the treated cells at each time-point were compared to time-matched untreated control cells in order to distinguish between constitutive and ligand-induced receptor trafficking.

**Figure 3:**
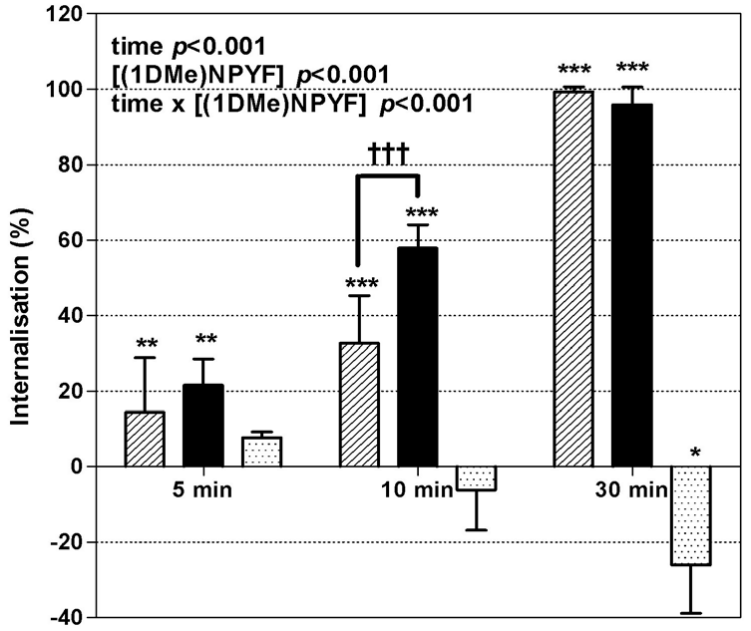
**The quantification of the cell surface fluorescence with FACS after DOR agonist challenge: the effect of (1DMe)NPYF on the agonist- induced internalization of DOR. **The cells were first treated with 100 nM DPDPE alone or 100 nM DPDPE + 100 nM (1DMe)NPYF or (1DMe)NPYF at 37°C for indicated times after which the cell surface receptors were detected with an anti-MYC-antibody and a fluorescent secondary antibody. 10000 cells/sample were analyzed with FACS. The combined data of five different experiments performed in duplicates is shown. The data is presented as the internalization percent that is calculated relative to the time-matched control cells (see Experimental section). The statistical significance was analyzed from the non-normalized raw data with two-way ANOVA (Bonferroni's post test, variance as SD, n = 4). *p < 0.05; **p < 0.01 and ***p < 0.001 shows statistical significance relative to the basal level (untreated time-matched cells), ^†††^*p *< 0.001 significant change in cell surface fluorescence between treatments. The dashed bars represent cells treated with 100 nM DPDPE alone, the solid bars cells treated with 100 nM DPDPE + 100 nM (1DMe)NPYF and the dotted bars represent the cells with 100 nM (1DMe)NPYF alone.

As seen in the FACS analysis, (1DMe)NPYF (100 nM) alone did not induce receptor internalization at any time-point tested (Figure 3). However, after 30 min treatment with the NPFF analogue somewhat higher fluorescence was detected on the cell surface as in the time-matched control cells. Already after 5 min treatment with 100 nM DPDPE or with 100 nM DPDPE and 100 nM (1DMe)NPYF the cell surface fluorescence was significantly different from the untreated control cells, indicative of ligand-induced internalization of the receptor. There was no significant difference between the treatments at this time-point although the double treatment with 100 nM DPDPE and 100 nM (1DMe)NPYF showed a slight tendency to decrease the surface fluorescence more. At 10 min time-point the treatment with 100 nM DPDPE together with 100 nM (1DMe)NPYF (57.9 % decrease in the cell surface fluorescence) caused significantly greater internalization of DOR than the treatment with 100 nM DPDPE alone (32.7 % decrease in the cell surface fluorescence) (Figure 3). At the 30 min time-point the cell surface fluorescence was at a significantly reduced level after both treatments but no significant difference was detected between the treatments (DPDPE alone and DPDPE together with (1DMe)NPYF).

### Characterization of Tb-DeltI and its binding on whole cells

**Figure 4:**
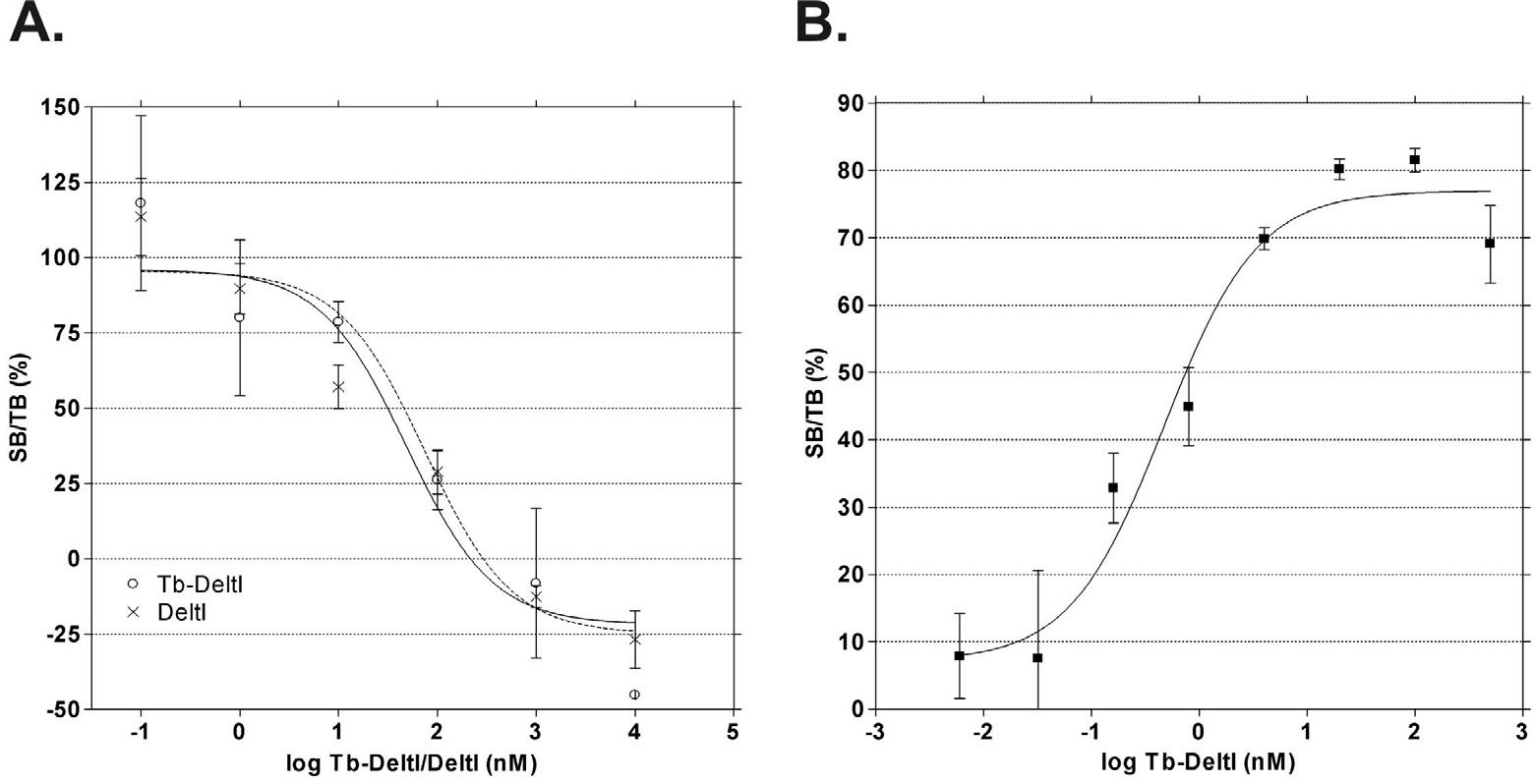
**The binding of Tb-DeltI to CHO/MYChDOR -cells. A. **Tb-DeltI can displace ^3^H-diprenorphine with similar affinity as unlabeled DeltI. In the competitive binding experiment 0.1 nM – 10 μM Tb-labeled DeltI or native DeltI was used to displace 6.86 nM^3^H-diprenorphine from the CHO/MYChDOR cell membranes. Tb-DeltI displaced the radioligand with a similar affinity as the unlabeled native DeltI. The solid line (curve fit) and the crosses represents the competitive binding for DeltI; the dashed line and the open circles for Tb-DeltI. The data is presented as the specific binding (SB) relative to the total binding (TB). The specific binding depicts the radioligand binding that can be displaced by the competing ligand (DeltI or Tb-DeltI). **B. **Tb-DeltI shows specific, saturable binding to DOR binding sites. The nonspecific binding of Tb-DeltI to CHO/MYChDOR -cells was determined in the presence of 1 μM DeltI. The binding of Tb-DeltI was saturable at a nanomolar range. The data is presented as the specific binding (SB) relative to the total binding (TB). The specific binding depicts the binding of Tb-DeltI that can be displaced by the unlabeled ligand.

To further investigate the effects of (1DMe)NPYF on DOR function a binding assay based on time-resolved fluorometry was developed. Tb-labeled DeltI was custom-made at Perkin Elmer Wallac. DeltI was labeled with a Tb-chelate to the Cys-modified N-terminus of the nascent peptide. To check whether the modification changed the affinity of the peptide, Tb-DeltI was tested in a classical radioligand displacement assay. Tb-DeltI displaced the ^3^H-diprenorphine from CHO/MYChDOR -cell membranes with similar affinity to the unlabeled DeltI (Figure 4A). Tb-DeltI bound to CHO/MYChDOR cell membranes dose-dependently and the binding was saturable at a nanomolar range (Figure 4B). The results with fixed whole cells showed similar binding characteristics as with the cell membranes, although the absolute values were below the ones observed with the cell membrane preparations. It was considered that the whole cell protocol can be used to study the effects of (1DMe)NPYF on the binding of Tb-DeltI on CHO/MYChDOR since only relative data between the treatments was desired. (1DMe)NPYF alone could not significantly displace Tb-DeltI. Next the effect of (1DMe)NPYF on the specific binding of Tb-DeltI was studied. The total binding of Tb-DeltI on the CHO/MYChDOR cells was determined in the presence of different concentrations of (1DMe)NPYF. The unspecific binding of Tb-DeltI was determined by 1 μM DeltI. (1DMe)NPYF modified the specific binding of Tb-DeltI to the CHO/MYChDOR cells. The K_D _for Tb-DeltI was only modestly affected by (1DMe)NPYF whereas 1 nM and 10 nM (1DMe)NPYF significantly decreased the B_MAX _for Tb-DeltI (Table 1). The higher concentrations of (1DMe)NPYF affected the B_MAX _only modestly.

### Inhibition of the forskolin induced accumulation of the cAMP

**Figure 5:**
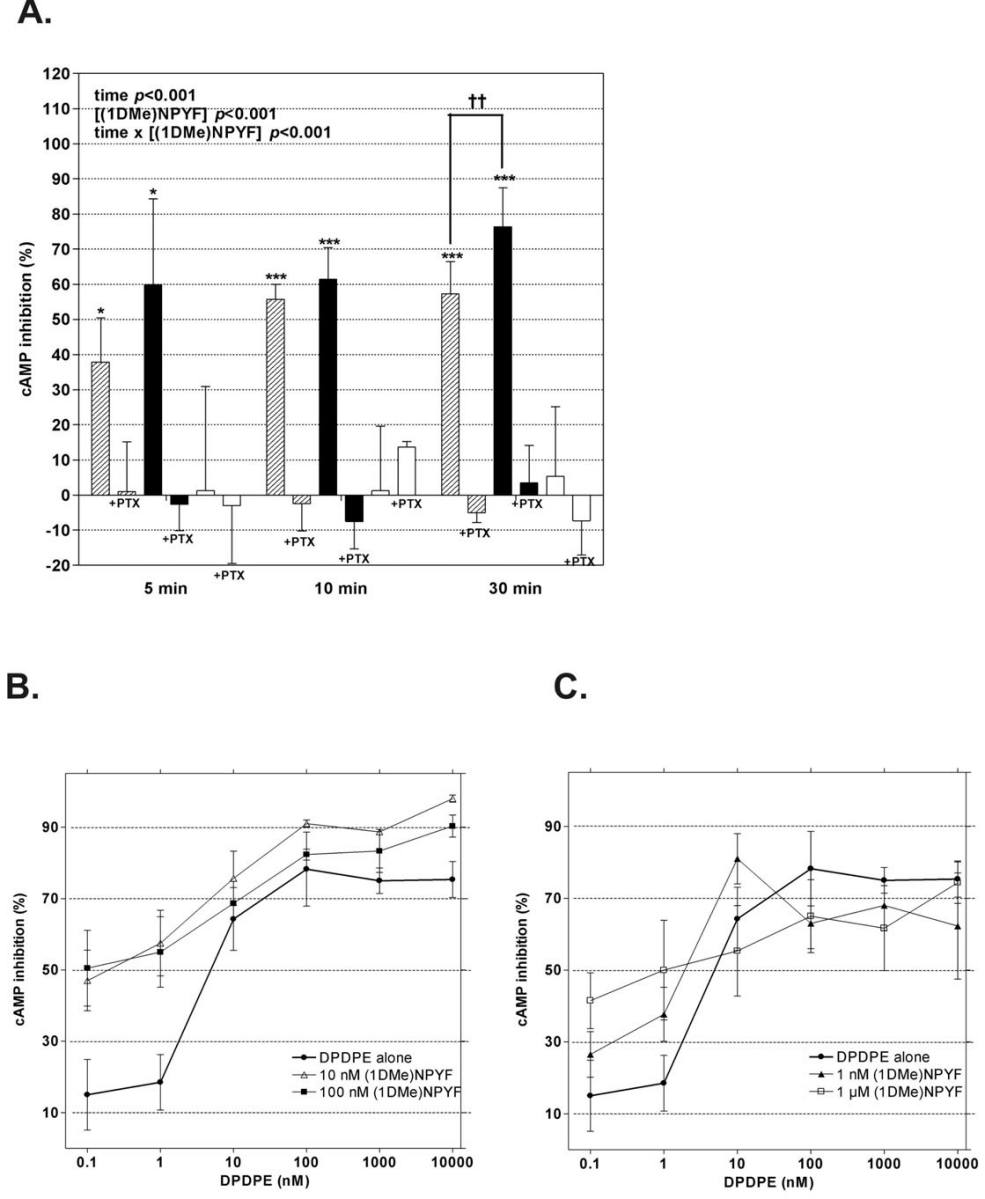
**(1DMe)NPYF enhances the DOR agonist induced inhibition of forskolin stimulated cAMP accumulation in the CHO-cells expressing MYChDOR. A. **The %-cAMP inhibition was calculated relative to the amount of cAMP in the cells treated with only forskolin. The cells were treated at 37°C for the indicated times with 10 μM forskolin together with 100 nM DPDPE (dashed bars), with 100 nM DPDPE + 100 nM (1DMe)NPYF (black solid bars) or with 100 nM (1DMe)NPYF (white solid bars). The bars next to each treatment at each time-point represent the cells pre-treated with pertussis toxin (+PTX). The statistical significance between treatments and time-points is shown in the figure (two-way ANOVA with Bonferroni's post test, variance as SD; significance relative to forskolin only treated cells ***p < 0.001; significance between treatments ^††^*p *< 0.01, n = 6). **B. **The effect of (1DMe)NPYF dose on the DPDPE-induced cAMP-inhibition. The cells were stimulated at 37°C for 30 min with 10 μM forskolin together with 0.1 nM – 10 μM DPDPE and 0 nM – 1 μM (1DMe)NPYF. (1DMe)NPYF caused a significant increase in the DPDPE-induced inhibition of cAMP (two-way Anova, variance as SD; DPDPE *p *< 0.001; (1DMe)NPYF *p *< 0.001, [DPDPE] × [(1DMe)NPYF *p *< 0.01, n = 3). The post-hoc test showed that 10 nM (*p *< 0.05) and 100 nM (*p *< 0.05) (1DMe)NPYF had a significant effect on the DPDPE induced cAMP inhibition. The solid circles with thick line represent the cells treated with 0.1 nM – 10 μM DPDPE alone, the open triangles cells treated with DPDPE together with 10 nM (1DMe)NPYF, and the solid squares cells treated with DPDPE together with 100 nM (1DMe)NPYF. **C. **1 nM (1DMe)NPYF and 1 μM had only minor effects on the DPDPE induced cAMP inhibition. The solid circles with thick line represent the cells treated with 0.1 nM – 10 μM DPDPE alone, the solid triangles cells treated with DPDPE together with 1 nM (1DMe)NPYF and the open squares DPDPE together with 1 μM (1DMe)NPYF.

DOR inhibits adenylate cyclase activity in numerous tissues and cell lines via coupling to G_i/o_-protein. We examined the ability of the ligand-activated MYC-tagged DOR to inhibit the forskolin stimulated cAMP accumulation in CHO-K1 cells. As expected, already 5 min treatment of the cells with the DOR agonist DPDPE (100 nM) reduced the level of forskolin-stimulated cAMP significantly (Figure 5A). Similarly, the treatment with DPDPE together with 100 nM (1DMe)NPYF at this time-point and at 10 min time-point inhibited the accumulation of cAMP. Although there was a slight tendency for (1DMe)NPYF to enhance the effect of DPDPE already at 5 min and 10 min time-points, the difference between the treatments was not significant. It was only after 30 min treatment that (1DMe)NPYF had a robust effect on the inhibition of cAMP induced by DPDPE (Figure 5A). At 30 min time-point the double-treatment with DPDPE + (1DMe)NPYF resulted in 76 % inhibition of forskolin stimulated cAMP accumulation in comparison to 57 % inhibition when treating the cells with DPDPE alone. (1DMe)NPYF alone did not have any significant effect on the amount of cAMP. Both the effect of DPDPE and DPDPE + (1DMe)NPYF was completely blocked by the pre-treatment with pertussis toxin (Figure 5A). (1DMe)NPYF was also able to enhance the inhibitory effect of other DOR agonists, e.g. DeltI, on the cAMP accumulation (data not shown), indicating that the modulatory effect of (1DMe)NPYF on the inhibition of cAMP accumulation is not agonist specific.

The effect of (1DMe)NPYF dose on the inhibitory action of DPDPE on the cAMP accumulation was also studied. The cAMP accumulation was measured at 30 min time-point. Above 100 nM DPDPE the cAMP inhibition reached saturation and addition of higher concentration of DPDPE did not result in further inhibition of forskolin stimulated cAMP accumulation (Figure 5B &5C). (1DMe)NPYF had a significant effect on the DPDPE-induced cAMP inhibition (two-way Anova, DPDPE *p *< 0.001; (1DMe)NPYF *p *< 0.001, [DPDPE] × [(1DMe)NPYF *p *< 0.01, n = 3). More specifically, 10 nM and 100 nM (1DMe)NPYF together with DPDPE enhanced the DOR agonist induced inhibition of cAMP accumulation significantly at all tested DPDPE concentrations (Figure 5B) whereas the lowest and highest tested concentrations of (1DMe)NPYF had hardly an effect on the cAMP accumulation (Figure 5C).

### Activation of MAP-kinase signaling pathway

**Figure 6:**
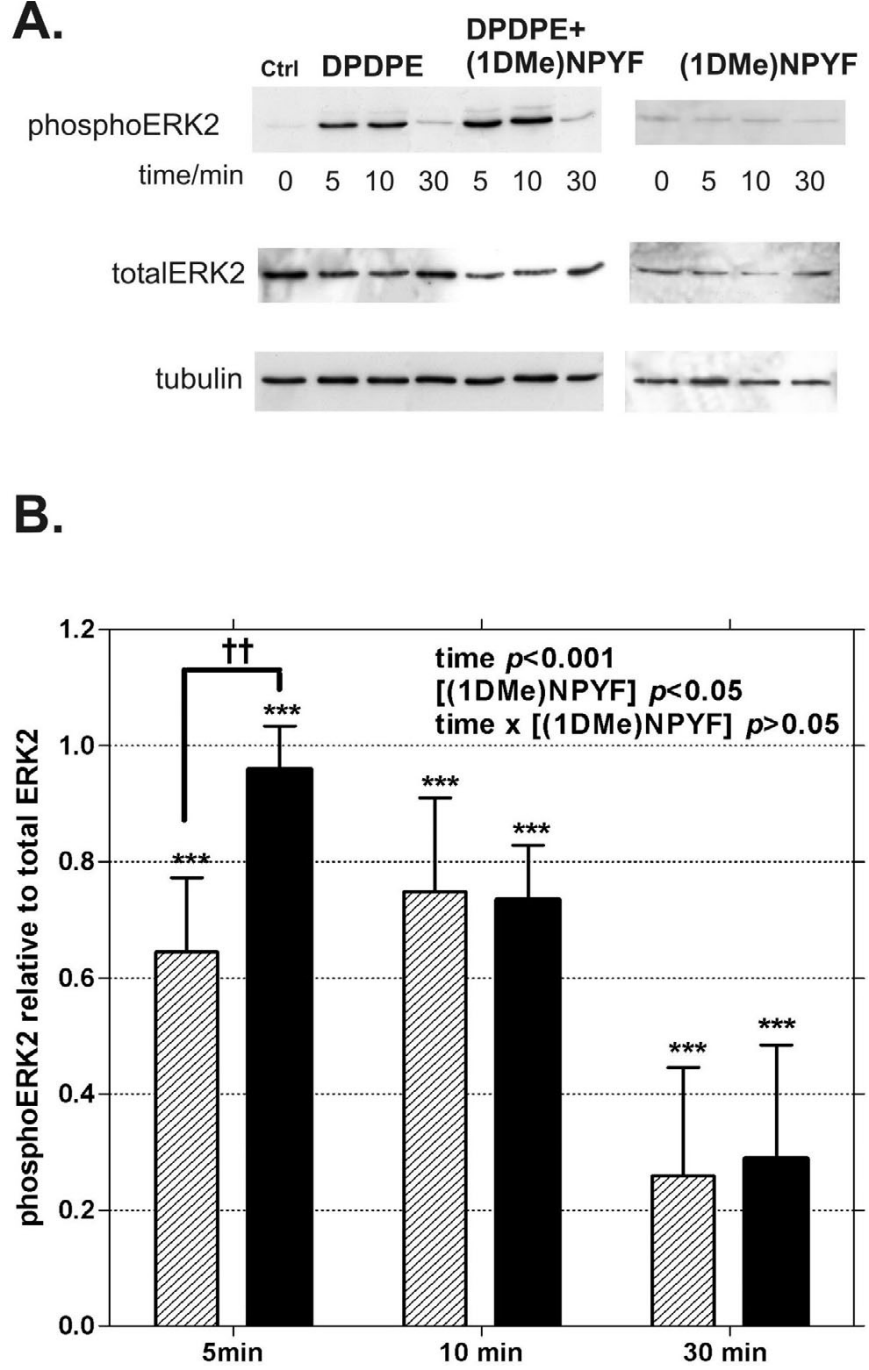
**DOR agonist induced phosphorylation of the MAP-kinase, ERK2, is increased by (1DMe)NPYF. A. **Total cell lysates (10 μg protein per lane) were detected with phosphoERK2 and total ERK2 specific antibodies. The equal loading of samples was controlled with anti-tubulin antibody. The cells were treated with 100 nM DPDPE alone (left panel), with 100 nM DPDPE together with 100 nM (1DMe)NPYF (middle panel) or with 100 nM (1DMe)NPYF (right panel) for 5, 10 or 30 min. (1DMe)NPYF alone could not induce the phosphorylation of ERK2 above the basal level. The data shown are representative figures of five independent experiments. **B. **The band intensities of phosphoERK2 were quantified and they are presented as relative band intensities with respect to the total ERK2. The dashed bars represent the cells treated with 100 nM DPDPE alone and the solid bars cells treated with 100 nM DPDPE and 100 nM (1DMe)NPYF. The statistical significance between time-points and treatments was analyzed with two-way Anova with Bonferroni's post-test, n = 5. Both of the treatments significantly induced the phosphorylation of the ERK2 above the basal level at all time-points (***p < 0.001). The difference between treatments was significant at the first time-point tested (^††^*p *< 0.01).

The activation of MAP-kinase p42^MAPK ^(ERK2) is characterized by the appearance of the phosphorylated form of the ERK2. The phosphorylated ERK2 was detected on the immunoblots after the cells were treated with DPDPE, (1DMe)NPYF or both for 0, 5, 10 and 30 minutes at 37°C. The basal level (t = 0 min) of phosphorylated ERK2 was at the detection limit of the antibody and only a very faint band could be detected (Figure 6A, left-most lane). When quantified, this band did not markedly differ from the background. The treatment of the cells with (1DMe)NPYF alone did not result in the activation of ERK2 (Figure 6A, right panel). Neither did any of the treatments significantly affect the total amount of ERK2. The total ERK2 was controlled from the immunoblots with an antibody detecting the unphosphorylated form of the kinase, which was compared to the amount of tubulin in each sample.

DPDPE (100 nM) activated the MAPK pathway as expected (Figure 6A and 6B). The peak in activation was observed after 10 minutes DPDPE-treatment followed by a decrease in the amount of the phosphorylated kinase. The addition of (1DMe)NPYF (100 nM) together with DPDPE (100 nM) significantly promoted the activation induced by DPDPE. The peak in activation with DPDPE + (1DMe)NPYF was detected already after 5 min in contrast to 10 min peak-activation time for DPDPE alone. At 10 min time-point there was no significant difference in the amount of phosphorylated ERK2 between DPDPE and DPDPE + (1DMe)NPYF-treated cells. However, at this time-point the level of phosphorylated ERK2 in the DPDPE + (1DMe)NPYF-treated cells had already started to decline whereas in the DPDPE-treated cells the level of phosphorylated ERK2 was still increasing when compared to the earlier and later time-points. In both DPDPE-treated cells and DPDPE + (1DMe)NPYF-treated cells after 30 minutes agonist stimulation the level of activated ERK2 had almost decreased back to basal values.

## Discussion

NPFF's role in physiological pain sensation and opioid analgesia is well characterized. Some of the NPFF's pain-related effects are mediated by the delta opioid receptor system [for review see ref. [8]]. In this study we examined the role of NPFF in the modulation of DOR-mediated cellular responses. We wanted to establish a simple model system to study the effect of NPFF analogue, (1DMe)NPYF, on the DOR function in the absence of NPFF receptors. We generated a cell line expressing N-terminally c-MYC-epitope tagged human DOR at close to physiological expression levels (see below). We also verified that the cells do not express functional NPFF receptors.

The expression level and binding affinity of the selected CHO/MYChDOR clone was within the same range as that reported for the well-studied NG108-15 neuroblastoma cell line [34] and for some native neuronal tissues e.g. rat striatum [35]. At this expression level the receptor trafficking and cellular signaling of the cells are expected to be normal but extreme over expression of a receptor might disrupt the functional properties of the cells. The cells responded to DOR ligands as expected in two different functional assays and the ligands also induced the internalization of DOR. In the basal state, some receptors were found in the internal parts of the cells, which is consistent with the previous findings for the delta opioid receptor [36]. Taken together, the pharmacological studies and confocal microscopic analysis together with functional assays suggested that the N-terminal modification of hDOR did not affect the ligand selectivity, functional coupling or trafficking of the expressed receptors.

DOR belongs to the large family 1 of GPCRs. We hypothesized that instead of, or in addition to operating via indirect mechanisms, NPFF could have a direct modulatory effect on DOR. Compounds that modulate the receptor activation have been described for other members of the receptor family [for a review see ref. [37]]. Modulators can potentate or block the agonist stimulation of the receptor in many ways, such as by changing the agonist affinity or stability of receptor-G-protein interaction [38-40].

Our FACS studies showed that in the presence of (1DMe)NPYF the agonist-induced internalization of DOR was enhanced. The increase in the receptor internalization by (1DMe)NPYF was most obvious after 10 min agonist exposure. The DOR antagonist naltrindole could completely block, not only the receptor activation by DOR agonists alone, but also the enhanced activation resulting from the co-application of (1DMe)NPYF with DOR agonists. This provides evidence that the observed effects were solely DOR-mediated. *In vivo *many of the analgesic effects of NPFF are blocked by both the non-specific opioid-antagonist naloxone and the DOR-specific antagonists naltrindole [4,44]. The differences between time-points and treatments in the FACS analysis were consistent and reproducible as assessed with statistical methods.

To study the effect of NPFF in DOR systems, we introduced a new tool to study the interactions between receptors and their ligands. A Tb-labeled DOR-ligand was used in a time-resolved fluorescence based binding assay and it proved comparable to the traditional radiolabel-based methods. Tb-DeltI could be applied to both cell membrane and whole cell assays. This new ligand and other related compounds open interesting possibilities to further study the ligand-receptor interactions, by using methods such as time-resolved FRET. Our results from the time-resolved fluorescence based binding assays indicate that NPFF can modulate the binding of DOR ligands to the receptor. Consistent with the earlier reports with spinal cord membranes [16], (1DMe)NPYF did not markedly bind to the classical DOR-binding site in the CHO/MYChDOR cells. Only micromolar concentration of (1DMe)NPYF could displace Tb-DeltI in whole cell and membrane binding assays to some extent. The binding experiments showed that (1DMe)NPYF affected the K_D _of Tb-DeltI only slightly, whereas the B_MAX _values were significantly decreased by low to modest concentrations of (1DMe)NPYF. It should be noted that the effects of (1DMe)NPYF on ligand binding observed in assays using a labeled agonist Tb-DeltI were partly verified by using a labeled antagonist, ^3^H-diprenorphine, as the ligand. In addition, while most of the experiments were performed with whole cells, a protocol that mimics the cellular environment for the natural receptor binding, the results were also to some extent confirmed with cell membrane preparations. This was to avoid the contribution of possible endogenous effector molecules present in whole cells.

The lack of change in the K_D _for opioid ligands implies that especially at low concentrations (1DMe)NPYF does not work through the classical opioid binding site of DOR. The dose-dependence of NPFF-agonist on the B_MAX _was bell-shaped. This could imply that the NPFF-analogue affects only some conformations of the receptor. Interestingly, also the effect of NPFF on prolactin release from rat pituitary cells show a bell-shaped dose-response curve [41]. However, the receptor involved in the prolactin release mechanism was not characterized. The NPFF-analogue had an effect on the B_MAX _at lower concentrations (1–10 nM) than on the signaling cascades studied. This could be related to the mechanism of the functional modulation of DOR by the NPFF-analogue. One possible mechanism could involve a modulatory binding site for NPFF on DOR. Modulatory binding sites have been described for other members of the GPCR family [37-40]. In line with our results, the GPCR modulators exert their effects commonly only in the presence of the receptor ligand [37]. In the future, the exact nature of interactions between DOR and NPFF will be further studied.

The modulatory effect of (1DMe)NPYF on DOR-mediated signaling was studied by measuring the activation of two differently regulated signaling cascades. The signaling studies suggested that (1DMe)NPYF alone did not affect DOR function but when applied together with a DOR agonist it significantly promoted the response of the activated signaling cascade. (1DMe)NPYF could enhance the DOR agonist induced inhibition of forskolin-stimulated cAMP accumulation, a signaling cascade primarily regulated by the G_i/o_α-subunit. The signaling via DOR was most strongly affected by (1DMe)NPYF at low to modest concentrations of DOR agonist. The cAMP inhibition resulting from double treatment with DPDPE + (1DMe)NPYF was greater than inhibition obtained by treatment with DPDPE alone at a broad DPDPE concentration range with various concentrations of the NPFF analogue. Also the maximal cAMP inhibition obtained with the double treatment was greater than that observed after treating the cells with a DOR agonist alone.

In addition to the regulation of cAMP levels, (1DMe)NPYF enhanced the DOR agonist-induced phosphorylation of ERK2, a member of MAP-kinase family. The activation ERK2 phosphorylation follows a series of activation steps initiated by the Gβγ-subunit. Interestingly, in the cells subjected to (1DMe)NPYF together with a DOR agonist an earlier peak in activation of ERK2 was observed than in cells treated with a DOR agonist alone. The finding that NPFF affects two differently regulated signaling cascades downstream from G-protein activation gives support for our hypothesis that NPFF works at the receptor level, prior to the G-protein activation. Both the DOR agonist-induced activation of receptor-mediated signaling and the enhanced activation caused by the addition of (1DMe)NPYF together with DOR agonist were completely blocked by PTX, giving evidence that NPFF delivers its effects via the same G-proteins as the DOR agonists binding to the classical binding site.

The enhancement of the DOR-mediated signaling could be due to the increased receptor trafficking. The accelerated internalization of the membrane-bound receptors could account for the enhanced signaling observed. The effect of (1DMe)NPYF on the internalization of DOR was transient and relatively small. There is some evidence that DOR ligands can affect the balance between receptors located on the cell membrane and receptors residing in the internal parts of the cells [45,46]. Petäjä-Repo et al. [45] have shown that DOR ligands can work as molecular chaperones, i.e. ligand binding to the receptor induces the transport of receptors to the membrane. It is likely that also the increased recruitment of the receptors from the internal pools to the cell membrane could account for the observed effects. Cahill et al. [46] showed that enhanced antinociceptive activity of DeltI is correlated with the recruitment of spinal opioid receptors from intracellular stores to the plasma membrane and that inflammation, a state that activates DOR -mechanisms, produces outward movement of intracellular DORs towards the plasma membrane. Therefore, the observed rate of internalization may be masked to some extent by the recruitment of the receptors from the internal pools. It should be noted that all the phenomena described above were observed with both the peptidic agonist DeltI and the cyclic agonist DPDPE. This implies that the effect is not between the agonists and (1DMe)NPYF but rather that (1DMe)NPYF modifies the receptor and alters its characteristics.

## Conclusion

Our results suggest that some of the NPFF's antinociceptive effects and interactions with opioid systems could at least in part be mediated directly via DOR. The agonist-induced internalization of DOR and the DOR mediated signaling are enhanced by NPFF, the specific binding of ligands to DOR is modified by NPFF. All changes observed in the different assays were in agreement with each other, although they had a bit different time-courses, and they all support the suggested direct interaction between NPFF and the DOR-ligand complex. The different time-course of events could be due to the various regulatory mechanisms involved. For example, the inhibition of the adenylate cyclase activity is directly regulated by the G_i/o_-protein α-subunit and the phosphorylation of ERK2 is mainly activated by the Gβγ subunit. The observed effects are also physiologically relevant, as published data suggest that opiate-induced analgesia is potentiated by NPFF [44], and the DOR antagonist naltrindole inhibits the NPFF-induced antinociception [4]. Although the modulatory effect of (1DMe)NPYF was moderate or in some cases quite small, it was consistently observed with all methods used in this study. As mentioned earlier, CHO-K1 cells do not express detectable levels of NPFF receptors. Therefore, DOR alone could be responsible for the modulatory effects of (1DMe)NPYF observed in this study. The results were obtained using a heterologous expression system and therefore they cannot be directly extended to *in vivo *conditions. Recent data based on opioid knockout mice show that DOR is mainly involved in the inflammatory and mechanical nociception [47]. NPFF expression is also significantly increased in inflammatory pain [3], and NPFF modulates descending inhibitory input to the wide-dynamic range neurons [8]. Some of the effects of NPFF in pathological pain and interactions with morphine may be mediated via DOR. This does not exclude e.g. the possible involvement of NPFF2R in pain mechanisms [18]. As two receptors for NPFF have been characterized, it seems likely that NPFF can have several functions depending on the site of action and availability of receptors. NPFF may be a multi-functional neuropeptide capable of affecting several neuronal systems through different receptors and signaling pathways, the effects depending on the other players present at the site action.

## Methods

### Materials

Cell culture medium components were from BioWhittaker (East Rutherford, NJ, USA), Sigma-Aldrich (St. Louis, MO, USA) or Gibco BRL/Invitrogen (Carlsbad, CA, USA). Blasticidin S HCl was from Invitrogen and FuGene 6 from Roche (Basel, Switzerland). [D-Pen(2,5)]enkephalin (DPDPE) and naloxone were purchased from Sigma. Deltorphin I (Y-D-AFDVVG-NH_2_) was from Bachem (Bubendorf, Switzerland). (1DMe)NPYF (D-YL-(NMe)-FQPQRF-NH_2_) was either custom-synthesized (Peptide Technologies, Gaithersburg, MD, USA) or from Bachem. Tb-labeled DeltI was custom made at Wallac (Perkin Elmer Wallac, Turku Finland) and all the reagents for the time-resolved fluorometry were purchased from Wallac. Mouse monoclonal anti-c-MYC antibody 9E10 and the c-MYC blocking peptide were from Sigma-Aldrich, rabbit polyclonal anti-c-MYC 9E10 antibody was from Santa Cruz Biotechnologies (Santa Cruz, California, USA), rabbit polyclonal C-terminal and N-terminal anti-NPFF2R antibodies and blocking peptides from Novus Biologicals (Littleton, CO, USA), rabbit anti-phospho-ERK1/2 antibody from Cell Signaling Technology (Beverly, MA, USA), mouse anti-ERK2 antibody from BD Transduction Laboratories (Becton Dickinson and Company, Franklin Lakes, NJ, USA), mouse anti-tubulin antibody from NeoMarkers (Fremont, CA, USA), goat anti-mouse Alexa 488 and goat anti-rabbit Alexa 488 antibodies from Molecular Probes (Eugene, Oregon, USA), goat anti-mouse horse radish peroxidase (HRP) and goat anti-rabbit-HRP conjugates from Bio-Rad (Hercules, California, USA). Nitrocellulose membrane was obtained from Schleicher & Schuell (Keene NH, USA). Enhanced chemiluminescence (ECL) reagents, ^125^I-(1DMe)NPYF and ^3^H-diprenorphine were from Amersham Pharmacia Biotech (Uppsala, Sweden). Restriction enzymes and other molecular biology products were purchased from Promega (Madison, WI, USA) or New England Biolabs (Beverly, MA, USA), PCR-primers were from Sigma Genosys (St. Louis, MO, USA). The CHO-K1/hNPFF2 cell membranes were from EuroScreen S.A. (Brussels, Belgium). The AcroWell filter plates were from Pall Life Sciences (NY, USA). General laboratory reagents were from Sigma-Aldrich or Merck (Darmstadt, Germany).

### Plasmid construction, transfection and cell culture

The human delta opioid receptor (a kind gift from Dr. Brigitte Kieffer) was N-terminally tagged with c-MYC-epitope (EQKLISEEDL). The coding sequence was amplified with primers that added the c-Myc epitope tag, a Kozak-sequence and an *Nhe*I-site immediately upstream of the hDOR and a *Bst*XI-site downstream of the receptor sequence. The PCR-product was inserted into *Nhe*I/*Bst*XI-site of the vector pcDNA6.0A (Invitrogen) and the construct was verified by sequencing. The human NPFF2R gene was cloned from human placental total RNA by RT-PCR. The full- length coding sequence was further subcloned into the pcDNA6.0A (Invitrogen) with similar methodology as that used for hDOR (instead of c-MYC tag, a FLAG tag was inserted N-terminally) and the construct was verified by sequencing.

The pcDNA6.0A-FLAGhNPFF2R construct was used for transient transfection of Chinese hamster ovary cells deficient in dihydrofolate reductase (CHO-K1, *dhfr*-). The plasmid was transfected into CHO-K1 cells with FuGene 6 reagent according to the supplier's instructions and 24 h post-transfection the cells were used for immunocytochemistry.

To create a stable cell line expressing c-MYC tagged human DOR, the pcDNA6.0A-MYChDOR vector construct was linearized with *Ssp*I and the linear DNA was used to transfect CHO-K1 cells. The stable transfection was performed using FuGene 6 reagent according to supplier's instructions. A stable clone expressing the receptor at the desired level was selected using FACS analysis. The cells were incubated with rabbit-anti-MYC in PBS supplemented with 1 % normal goat serum (v/v) on ice, washed with ice-cold PBS and further incubated with the goat anti-rabbit Alexa 488 antibody in PBS on ice. After washes with ice-cold PBS the cells were suspended in PBS supplemented with 10 % fetal calf serum (v/v) and sorted. After expansion the obtained cells were plated onto 96-well plate and clones originating from a single cell were selected. The clones were analyzed for the receptor expression with immunocytochemistry and radioligand binding and the functionality of the heterologously expressed receptor was tested with MAPK and cAMP -assays.

The expression of NPFF receptors in CHO-K1 cells was tested with reverse-transcription polymerase chain reaction (RT-PCR). Total RNAs from wild type CHO-K1 cells and the CHO-K1 clone expressing MYC-tagged DOR were isolated with RNAwiz protocol (Ambion). Total RNA from human placenta and human medial hypothalamus were isolated and were used as positive controls in RT-PCR. cDNA from the total RNA was produced with The Superscript First-Strand Synthesis System for RT-PCR (Invitrogen). The expression of NPFF receptors was analyzed with several primer pairs specific for either human or mouse receptors (the receptors from hamster have not been cloned). Sequences for the primers specific for the human NPFF2R were: 5'-GAT TGG TCC AGG GAA TAT CTG TC-3' and 5'-CAG TGT GCA AAA GGG TAG ATG TAG-3' or 5'-GGA TGG CCA TTT GGA AAC-3' and 5'-CCA ATC CTT CCA TAC ATG-3'. Sequences for the primers specific for the human NPFF1R were 5'-CGA CAA TGC CAC ATG CAA GAT GAG-3' or 5'-CGC AAC CGC TCC TAC CCT CTC TAC-3' together with 5'-AGG GGA AGG CGT AGA CGG TGA C-3'. Corresponding mouse specific primers were also used in the analysis.

CHO-K1 cells were cultured in Dulbecco's modified Eagle's medium (DMEM) supplemented with 10 % (v/v) FBS, 4.5 g/litre glucose, 100 U/ml penicillin, 0.1 mg/ml streptomycin, 1 mM glutamine and 1 × HT-supplement. For the transfected cells the selection pressure was maintained by adding 0.5 μg/ml Blasticidin S HCl to the culture media. The cells were kept at 37°C in 5 % CO_2_/humidified air and the culture medium was changed every 3–4 days. The cells were sub cultured in ratio of 1:5–1:10 and only cells undergone fewer than 20 passages were used for the experiments.

### Immunocytochemistry and confocal microscopy

The cells were collected, plated onto 18 mm × 18 mm cover slips and cultured o/n. After o/n incubation the cells were rapidly cooled on ice, the medium was removed and the cells were washed two times with ice-cold PBS. The cells were fixed with 4 % PFA (w/v) for 30 min at +4°C and washed twice with PBS. The fixed cells were first incubated with 1 % normal goat serum (v/v); with or without 0.05 % saponin (w/v) in PBS for 30 h at +4°C and then o/n with 1:5000 dilution of mouse anti-c-MYC/1:2000 dilution of rabbit anti-NPFF2R antibody in 1 % normal goat serum (v/v) in PBS. After washes with PBS the cells were incubated with 1:1000 dilution of goat anti-mouse/anti-rabbit Alexa 488 antibody in PBS for 1 h at +4°C. They were then washed with PBS and the cover slips were mounted onto objective glasses.

The slides were analyzed with a Leica TCP-SC laser scanning microscope system with Ar-Kr laser (Omnichrome, Melles Griot, Carlsbad, CA, USA). The images were acquired and processed with Leica TCS NT/SP Scanware software. The excitation and emission wavelengths used were for the Alexa 488 secondary antibody excitation 488 nm and emission 492–540 nm. The samples were scanned with identical settings taking images every 0.5 μm. The PMT values for the laser scanner were set with a negative control slide, i.e. autofluorescence was eliminated. The image representing the mid-nuclear level of the scanned sample was taken from the image stack for a comparative analysis between time-points and treatments. The scanned images were further analyzed and the figures for the publication were produced with Adobe Photoshop 6.0 (Adobe Systems, San Jose, CA, USA) and/or Corel Draw 9.0 software (Corel, Ottawa, Canada). Each image was again treated similarly and the figures were not manipulated in any way. Representative images were chosen for the Figures 1 and 2.

### Flow cytometry

The CHO/MYChDOR cells were collected and suspended into the culture medium. The cells were treated with drugs in suspension as for the immunocytochemistry for the indicated times. After the incubation, the membrane trafficking was stopped by rapidly chilling the cells on ice and removing the medium. After this the cells were kept on ice. The cells were washed with PBS and stained in PBS-suspension with rabbit anti-MYC antibody (1 % normal goat serum (v/v) used to block unspecific binding), followed by the detection with a fluorescent secondary antibody. Dead cells were detected with isopropidium iodide. Cells were analyzed with FACScan flow cytometer (Becton Dickinson and Co., Mountain View, CA, USA). Data from 10000 cells were collected for each run and each sample was run in replicate or triplicate.

The internalization percent was calculated as:  where F(ctrl) in the fluorescence of the time-matched control cells and the F(sample) is the fluorescence measured from the drug-treated cells.

### Determination of intracellular cAMP levels

50000 cells/well seeded onto 96-well plates and incubated o/n. In the case of pertussis toxin pre-treatment, the cells were incubated 16–20 h with 100 ng/ml PTX before the exposure for drugs. The cells were incubated for one hour with serum-free medium at 37°C; then 0.3 mM 1-Methyl-3-isobutylxanthine (IBMX) and 0.3 mM indomethazine were added and the cells were further incubated for 10 min. After this 10 μM forskolin was added with or without the drugs (DPDPE, (1DMe)NPYF or DPDPE + (1DMe)NPYF). The cells were incubated for the indicated times at 37°C, the reaction was stopped by adding lysis buffer (supplied by the kit) and cooling the cells on ice. The amount of cAMP was determined from the cell lysates with Delfia cAMP kit (Perkin Elmer Wallac, Turku, Finland). The time-resolved fluorescence was measured with Victor^2 ^Multilabel counter (Perkin Elemer Wallac, Turku, Finland).

### Phosphorylation of MAPK

25000 cells/well were seeded onto 24-well plates and incubated o/n. The medium was replaced with serum-free medium and the cells were incubated for one hour at 37°C. Serum-free medium containing 100 nM DPDPE or 100 nM (1DMe)NPYF or 100 nM DPDPE + 100 nM (1DMe)NPYF (end concentrations) was added and the incubation was continued for the times indicated. The reaction was stopped by removing the medium and adding modified RIPA-lysis buffer (PBS, pH 7.6; 1 % Nonidet P-40 (v/v); 0.5 % sodium deoxycholate (w/v); 0.1 % SDS (w/v), 10 mM NaF; 0.4 mM PMSF, 10 μg/ml aprotinin, 10 μg/ml leupeptin, 2 mM Na_2_VO_5_; 0.2 mM bestatin). Cells were lysed on ice for 15 min and the lysates were collected. The protein content was determined with the Bradford method.

10 μg of protein was analyzed on 10 % SDS-PAGE gel and the gel was blotted onto nitrocellulose membrane. The membrane was blocked with 5 % fat-free dry milk (w/v) in TBS-T (10 mM Tris/HCl pH 7.6; 150 mM NaCl; 0.05 % Tween 20 (v/v)). It was then incubated with 1:2000 dilution of rabbit anti-phospho-ERK1/2 antibody in the blocking buffer o/n at 4°C, washed with TBS-T, incubated for one hour at room temperature with 1:3000 dilution of goat anti-rabbit-IgG conjugated to HRP in the blocking buffer. After washes, the membrane was detected with the ECL-protocol. The band intensities were measured with AlphaDigiDoc1000 documentation system (Alpha Innotech, San Leandro, CA, USA).

After stripping with 0.2 M glycine; 0.5 M NaCl, pH 2.5 the membrane was similarly stained with mouse anti-ERK2 (1:5000 dilution) and mouse anti-tubulin (1:1000 dilution) antibodies to determine the total amount of ERK2 and to control the equal loading of samples, respectively. Goat anti-mouse-IgG conjugated to HRP (1:3000 dilution) was used as the secondary antibody.

### Preparation of cell membranes

The cells were collected with PBS + 0.2 % EDTA (w/v) and washed with cold PBS. The CHO/MYChDOR or CHO-K1 cells were suspended into ice-cold 50 mM Tris/HCl, 5 mM EDTA, pH 7.5 and homogenized with Potter S-homogenisator on ice. The homogenate was centrifuged 47800 × g at 4°C and the pellet was suspended into the Tris/EDTA buffer and the homogenization/centrifugation was repeated. The pellet was suspended into cold binding assay incubation buffer and the protein content was measured with the Bradford method.

### Radioligand binding on cell membranes

The binding experiments were performed as described in [21]. 25 μg or 50 μg of crude membrane preparation prepared as described above was used per assay. As positive control membrane preparation from EuroScreen S.A. (Brussels, Belgium) was used. For the saturation binding assay the membranes were incubated for one hour at room temperature with 0.2–10 nM ^3^H-diprenorphine in 50 mM Tris/HCl, pH 7.5 or 0.002 – 0.2 nM ^125^I-(1DMe)NPYF in the binding buffer described in [21]. The non-specific binding was determined with 10 μM naloxone for ^3^H-diprenorphine and with 1 μM (1DMe)NPYF for ^125^I-(1DMe)NPYF. In the displacement assays the effect of 0.1 nM – 10 μM DPDPE and/or (1DMe)NPYF on 6 nM ^3^H-diprenorphine binding was determined. Otherwise, the assay conditions were as for the saturation assay.

### Tb-DeltI binding on cell membranes and whole cells

6 μg of crude membrane preparations or whole cells (25 000 cell/well) that were plated one day before experiment and fixed with 4 % PFA for 10 min at room temperature, were used in the saturation binding experiments. The whole cell assays were carried out on normal cell culture plates and the assays with membrane preparations on special filter plates (Pall Life Sciences). The cells or membranes were incubated in 50 mM Tris/HCl pH 7.5; 2.5 mM MgCl_2_; 60 mM NaCl; 25 μM EDTA, 0.2 % BSA (w/v) for 90 min at room temperature with 0.01 – 781.25 nM Tb-DeltI in the presence or absence of 1 or 5 μM unlabeled DeltI. To study the effect of (1DMe)NPYF on the Tb-DeltI binding, 1–1000 nM (1DMe)NPYF was added together with unlabeled DeltI. After the incubation the unbound Tb-DeltI was removed by washing the plates four times with 50 mM Tris/HCl pH 7.5; 2.5 mM MgCl_2_. Vacuum pump system was used to wash the filter plates. Since the chelate-bound Tb-ion is nonfluorescent, it needed to be released from the complex. In order to release Tb-ion from the chelate 150 μl Delfia enhancement solution was added to the wells and the plates were incubated on a shaker for 15 min. Thereafter, 40 μl Delfia enhancer that changes the pH of the assay solution appropriate for Tb-fluorescence was added, and the plates were further incubated for 5 min on a shaker. The time-resolved fluorescence of the released Tb-ion was measured with Victor^2 ^Multilabel counter (Perkin Elemer Wallac, Turku, Finland).

### Statistics

All the experiments were repeated at least three times with three replicates in all assays. Prism GraphPad Softwear version 4.0 (GraphPad Softwear, San Diego, CA, USA) was used for all statistical analysis. Two-way Anova with Bonferroni's post-test or one-way Anova with Bonferroni's multiple comparison test was used to assess the statistical significance of the binding assay, cAMP-assay, MAPK-assay and FACS-analysis results. When a parametrical test was used the analysis was done on the raw, non-normalized data.

## List of abbreviations

(1DMe)NPYF, D-YL-(NMe)-FQPQRF-NH_2_; CHO, Chinese hamster ovary; DeltI, deltorphin I; DOR, delta opiate receptor; DPDPE, [D-Pen(2,5)]enkephalin; ERK, extra cellular signal-regulated kinase; GPCR, G-protein coupled receptor; IBMX, 1-Methyl-3-isobutylxanthine; NPFF, neuropeptide FF; MAPK, mitogen-activated protein kinase; PFA, paraformaldehyde; PTX, pertussis toxin

## Authors' contributions

M-LÄ participated in planning the experiments, executed them, and wrote the manuscript. PP participated in planning of the experiments, and interpretation of the results, and obtained funding. Both authors revised and accepted the manuscript.

## Figures and Tables

**Table 1:** The effect of (1DMe)NPYF on the specific binding of Tb-DeltI on CHO/MYChDOR cells. The K_D _and B_MAX _values for Tb-DeltI binding were determined in the presence of different concentrations of (1DMe)NPYF. Native DeltI (1 μM) was used to determine the nonspecific binding. Statistical significances were calculated relative to specific binding in the absence of (1DMe)NPYF (one-way Anova, ***p < 0.001, n = 3). The values in the table are from whole cell assays and therefore provide only relative information about the K_D _and B_MAX _values between treatments.

**(1DMe)NPYF (nM)**	**K_D_(nM)**	B_MAX_(pmol/cell)*
**0**	75.8 ± 8.1	1.60 ± 0.06
**1**	52.1 ± 6.5	0.70 ± 0.08 ***
**10**	55.5 ± 7.6	0.89 ± 0.06 ***
**100**	82.5 ± 3.9	1.42 ± 0.11
**1000**	80.5 ± 8.2	1.49 ± 0.10

*Relative B_MAX_-values from whole cell binding experiments; calculated as pmol/cell.
